# Protective Effect of Silymarin and Gallic Acid against Cisplatin-Induced Nephrotoxicity and Hepatotoxicity

**DOI:** 10.1155/2022/6541026

**Published:** 2022-04-16

**Authors:** Duygu Doğan, İsmet Meydan, Ahmet Ufuk Kömüroğlu

**Affiliations:** Van Yuzuncu Yil University, Vocational School of Health Services, Van, Turkey

## Abstract

**Objective:**

This study aimed to investigate the effects of gallic acid and silymarin against nephrotoxicity and hepatotoxicity caused by cisplatin.

**Materials and Methods:**

In the study, 56 Wistar Albino rats were equally divided into eight groups. Group 1 was the control group; group 2 was the group receiving cisplatin; group 3 was the group receiving cisplatin + gallic acid; group 4 was the group receiving cisplatin + silymarin; group 5 was the group receiving cisplatin + silymarin + gallic acid; group 6 was the group receiving silymarin; group 7 was the group receiving gallic acid; group 8 was the group receiving gallic acid + silymarin. AST, ALT, urea, creatinine, albumin, globulin, and total protein levels were measured at the end of the study. Superoxide dismutase (SOD), catalase (CAT), malondialdehyde (MDA), glutathione (GSH), and 8-hydroxy-2′-deoxyguanosine (8OH-dG) levels were measured in kidney and liver tissues. Additionally, histopathological evaluations of the tissues were also performed.

**Results:**

In kidney and liver tissues, cisplatin significantly increased MDA and 8-OHdG levels compared with treatment groups (*p* < 0.05). Silymarin-treated group significantly increased the SOD activity and GSH amount in the liver tissue compared with the cisplatin-treated group (*p* < 0.05). Gallic acid significantly increased CAT activity compared with the cisplatin-treated group (*p* < 0.05). It was determined that the cisplatin-treated group significantly decreased CAT and SOD activity compared with the control group (*p* > 0.05). Gallic acid showed a significant increase in CAT and SOD activity in kidney tissue compared with the cisplatin-treated group (*p* < 0.05).

**Conclusion:**

As a result, it was observed that gallic acid silymarin had a protective effect on cisplatin-induced nephrotoxic and hepatotoxic effects.

## 1. Introduction

The formation of factors such as air pollution, nutritional disorders, tobacco, and alcohol consumption in recent years has led to an increase in free radicals in the body and the emergence of many diseases such as cancer [1]. Cells are protected against these harmful effects by antioxidant systems. The protection of antioxidant capacity is very important for the continuation of vitality, thus enabling experimental and clinical research [2].

It is known that plants and fruits contain antioxidant components that contribute to body defense against oxidative stress [3, 4]. In line with the studies, it has been observed that antioxidants have diabetes, antibacterial, antifungal, antiviral, antitumoral, antiulcer, and anticarcinogenic effects [5]. The use of herbal medicines has increased due to their protective effects on organ toxicity. For a long time, silymarin has been a natural solution to protect against toxic substances, especially in liver diseases. Besides its antifibrotic, immunomodulating, and anti-inflammatory effects, it has been found to have antioxidant properties by scavenging free radicals [6].

Silymarin, which is used as a strong antioxidant thanks to the phenolic structures it contains, draws attention with its protective feature against toxic effects. Silymarin is called a hepatoprotectant with a short half-life and rapid conjugation in the liver [7]. In a study with cisplatin, it was observed that it has antioxidant effects by increasing the gene expression of antioxidant enzymes on kidney tissue and provides a protective effect against damage. It has been observed that positive results have been obtained in studies with the effect of silymarin against both hepatotoxicity and nephrotoxicity [8].

Gallic acid, which is a phenolic acid found in many foods and herbs, shows itself as a powerful antioxidant. It has been determined that gallic acid has antioxidant properties by scavenging free radicals directly [9]. In the research conducted due to the side effect of gallic acid caused by cisplatin, it was stated that it had a remarkable protective effect against DNA damage [10].

The drugs used have many side effects. Therefore, many studies have been carried out to minimize or eliminate these side effects. Although cisplatin is a widely used chemotherapeutic agent, its use has been limited due to its side effects [11]. Possible side effects include hepatotoxicity, ototoxicity, emetogenesis, myelosuppression, and spermiotoxicity. Nephrotoxicity and hepatoxicity were the most common side effects of cisplatin, especially with the inhibition of antioxidant enzymes and proteins by creating oxidative stress [12]. For this reason, it is important for studies to investigate the protective effects of various antioxidant-containing substances against these effects.

The fact that both silymarin and gallic acid are strong antioxidants and have protective effects in line with the studies led us to investigate their effects against cisplatin-induced nephrotoxicity and hepatotoxicity. Since there is no previous study with these two components, our research is an original study.

## 2. Material and Method

### 2.1. Live Material

In the study, 56 male Wistar albino rats, 8–10 weeks old, weighing 150–250 gr, obtained from Van Yüzüncü Yıl University Experimental Medicine Application and Research Center were used. During the experiment, the rats were housed in cages with 12 hours of darkness/lighting in rooms with a temperature of 22 ± 2°C, with constant feed and fresh water in front of them. The Animal Experiments Local Ethics Committee of Yüzüncü Yıl University approved the study (28.05.2020, 2019/05–03).

### 2.2. Determination of Working Groups

In the study, 56 male Wistar albino rats were randomly selected, with seven rats in each group, and eight groups including the control group were formed as follows: group 1 was the control group; group 2 was the group receiving cisplatin; group 3 was the group receiving cisplatin + gallic acid; group 4 was the group receiving cisplatin + silymarin; group 5 was the group receiving cisplatin + silymarin + gallic acid; group 6 was the group receiving silymarin; group 7 was the group receiving gallic acid; group 8 was the group receiving gallic acid + silymarin.

### 2.3. Gallic Acid, Silymarin, and Drug Administration

During the seven-day study, 8 mg gallic acid and silymarin per kg were dissolved in water and administered via gastric gavage [13]. On the fourth day of the study, cisplatin (cisplatin DBL 10 ml 10 mg vial, Orna İlaç Sanayi, Turkey) was administered as a single dose of 3 cc per rat intraperitoneally (ip) [14].

### 2.4. Collection of Blood Samples

After the seven-day experiment, ketamine (75 mg/kg) and xylazine (10 mg/kg) were administered ip to the rats that were fasted for 12 hours. Intracardiac blood samples were taken into vacuum tubes with and without anticoagulants.

### 2.5. Collection of Liver and Kidney Tissue Samples

After the blood samples were taken, the kidneys and livers of the rats, which were sacrificed by the bloodless method, were removed. It was stored at −80°C until analysis.

### 2.6. Histopathological Evaluation

After the rats were anesthetized with ketamine and xylazine, the kidneys and liver tissues were removed. The excised tissues were fixed in buffered formaldehyde. Then, after going through routine histological tissue follow-up stages, it was embedded in paraffin. Sections of 5 *μ*m thickness, taken with a microtome from kidney and liver tissues embedded in paraffin blocks, were stained with hematoxylin-eosin dye and examined under a light microscope (Olympus BX53, Tokyo, Japan). Photographing was done with the Olympus DP74 microscope camera and Olympus cellSens software. For histopathological evaluation, a mean of 15 fields for each animal in the groups was evaluated by random sampling. The findings were evaluated semiquantitatively according to the degree of damage observed in the examined regions. Accordingly, scores were as follows: normal tissue: −; minor damage: + (damage <25%); minor damage: ++ (25–50%); medium damage: +++ (50–75%); severe damage: +++ (damage >75%).

### 2.7. Analyzes in Kidney and Liver Tissue

#### 2.7.1. Preparation of Tissue Homogenate

Phosphate buffer (pH: 7.2–7.4) was added to the kidney and liver tissues at 10 times their weight. It was homogenized with the aid of a homogenizer. Care was taken to ensure the cold chain at all stages of the experiment. The homogenates were centrifuged at 2000–3000 rpm, +4°C for 20 minutes [15]. GSH level (BT Lab no.: E1101 Ra), CAT (BT Lab no.: E0869 Ra), and SOD (BT Lab no.: E0168Ra) enzyme activities and 8-OHdG level (BT Lab no.: E0031Ra) using commercial kit by ELISA method measurements were determined on the ELISA device (BioTek ELx800).

### 2.8. Statistical Analysis

SPSS v.20 (Chicago, IL, USA) package program was used for statistical analysis. All data were expressed as mean ± standard deviation. Statistical analysis of the groups was performed using the one-way ANOVA followed by post hoc multiple comparisons (Tukey's test) for comparative analysis between the groups. *p* < 0.05 was considered statistically significant.

## 3. Results

### 3.1. Biochemical Evaluation

Serum GSH, CAT, SOD, and 8OH-dG levels were compared between the groups (Table 1). Compared with the control group, a significant decrease in GSH level and SOD activity and a significant increase in 8-OHdG levels were detected in the cisplatin group. Compared with the cisplatin group, it was determined that the GSH level increased in the cis + gal, cis + sly, and cis + sly + gal groups and the CAT activity in the cis + sly + gal group (*p* < 0.05). It was determined that the level of 8-OHdG decreased significantly in the cis + gal group compared with the cisplatin group (*p* < 0.05).

The levels of GSH, CAT, SOD, MDA, and 8OH-dG in liver tissue were discussed between groups using the data in Table 2. Compared with the control group, the MDA and 8OH-dG levels of the group receiving cisplatin were significantly higher (*p* < 0.05); the CAT and SOD activities and GSH levels of the group receiving cisplatin were significantly lower (*p* < 0.05). MDA and 8OH-dG levels were significantly lower in the cis + sly + gal, cis + sly, and cis + gal groups compared with the group receiving cisplatin (*p* < 0.05). SOD activity was significantly higher in the cis + sly + gal, cis + sly, and cis + gal groups compared with the group receiving cisplatin (*p* < 0.05). Compared with the control group, the MDA level of the cis + sly + gal group was significantly higher (*p* < 0.05). SOD activity of the cis + sly + gal group was significantly higher than the group receiving cisplatin (*p* < 0.05). In these groups, CAT and GSH levels were high but not significant (*p* > 0.05).

The levels of GSH, CAT, SOD, MDA, and 8OH-dG in kidney tissue were discussed between groups using the data below (Table 3). Compared with the control group, the MDA and 8OH-dG levels of the group receiving cisplatin were significantly higher (*p* < 0.05), and the CAT and SOD activities of the group receiving cisplatin were significantly lower (*p* < 0.05). However, GSH level was low, but this value was not significant (*p* > 0.05). MDA and 8OH-dG levels were significantly lower in the cis + sly + gal, cis + sly, and cis + gal groups compared with the group receiving cisplatin (*p* < 0.05). SOD activity was significantly higher in the cis + sly + gal, cis + sly, and cis + gal groups compared with the group receiving cisplatin (*p* < 0.05). However, GSH level was low, but this value was not significant (*p* > 0.05).

Compared with the control group, the RBC and HGB levels of the group receiving cisplatin were significantly higher (*p* < 0.05), and the PLT value was significantly lower (*p* > 0.05). Compared with the group receiving cisplatin, the WBC, RBC and HGB values of the cis + sly + gal group were significantly higher, and the PLT value was significantly lower (*p* > 0.05) (Table 4).

Compared with the control group, the serum urea and creatinine levels of the group receiving cisplatin were significantly higher (*p* < 0.05), and albumin, total protein, testosterone, urea, AST levels were low, but this decrease was not significant (*p* > 0.05). Compared with the group receiving cisplatin, serum creatinine level was found to be lower, and globulin level was significantly higher in the cis + sly + gal group (*p* < 0.05). Compared with the group receiving cisplatin, AST, urea, and creatinine levels were significantly lower in the cis + gal group (*p* < 0.05) (Table 5).

### 3.2. Histopathological Evaluation

#### 3.2.1. Kidney

**Figure 1:**
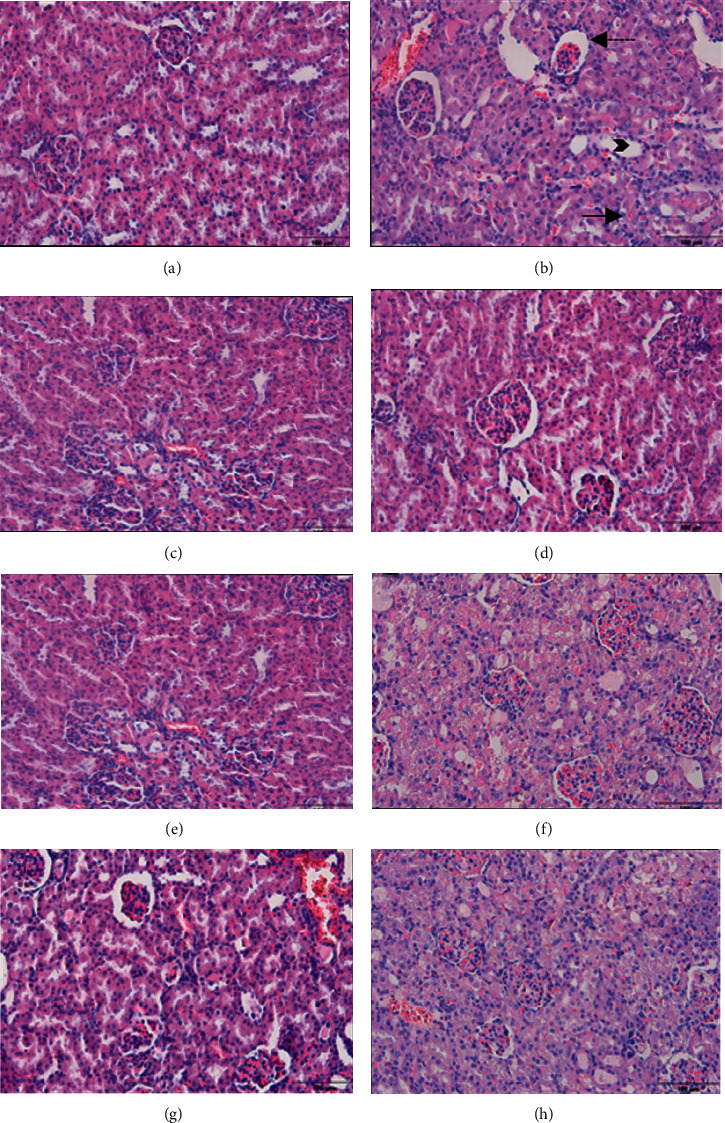
Light microscopic images of kidney tissue. (a) Control, (b) cis, (c) gal, (d) sly, (e) gal + sly, (f) cis + gal, (g) cis + sly, and (h) cis + gal + sly. Straight arrow: tubular degeneration. Dashed arrow: filtration gap width. Arrowhead: tubular dilation.

In the light microscopy examination, it was observed that the kidney tissues of the control, gal, sly, and gal + sly groups were in normal histological structure (Figure 1). Severe tubular cell degeneration, tubular dilatation, and enlargement of the Bowman's capsule space were observed in the kidney tissue of rats receiving cisplatin. Middle tubular cell degeneration, tubular dilatation, and expansion of Bowman's capsule space were observed in the kidney tissue in the cis + gal and cis + sly groups, and mild tubular cell degeneration, tubular dilatation, and enlargement of the Bowman's capsule space were observed in the kidney tissue in the cis + gal + sly group.

#### 3.2.2. Liver

**Figure 2:**
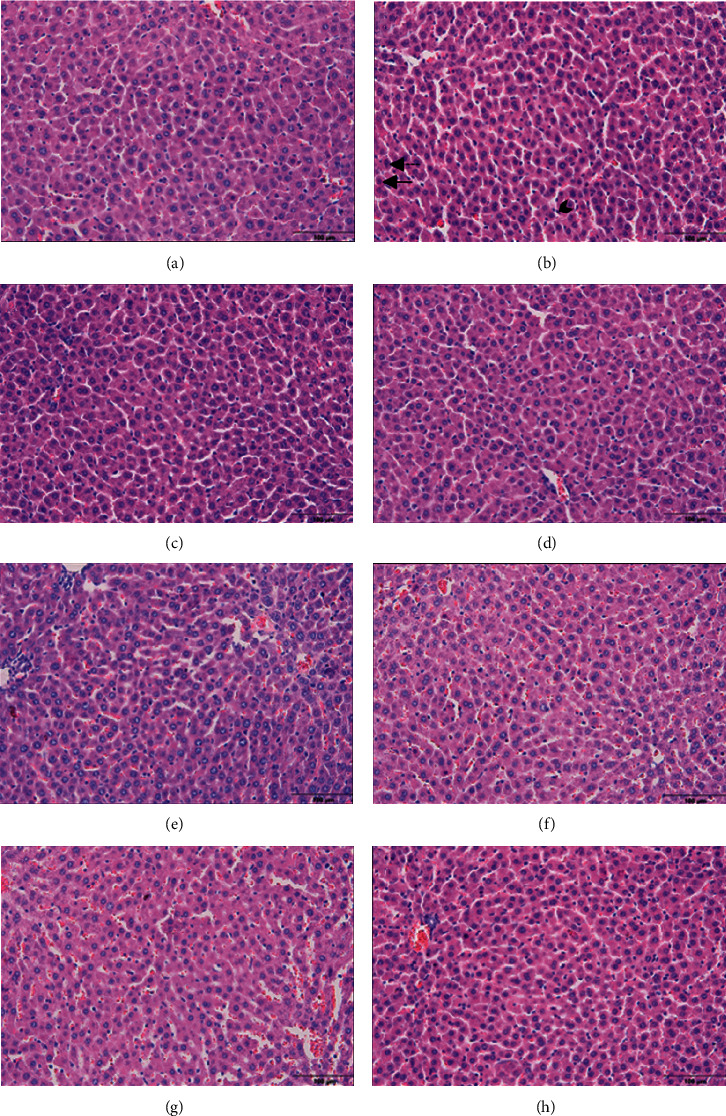
Light microscopic images of liver tissue. (a) Control, (b) cis, (c) gal, (d) sly, (e) gal + sly, (f) cis + gal, (g) cis + sly, and (h) cis + gal + sly. Straight arrow: pyknotic hepatocytes. Dashed arrow: karyolytic hepatocytes. Arrowhead: Kupffer cell.

In the light microscopy examination, it was observed that the liver tissue of the control, gal, sly, and gal + sly groups had a normal histological structure (Figure 2). In rats receiving cisplatin, hepatocytes with severe pycnotic and karyolytic nuclei were observed in the liver tissue of the rats receiving cisplatin. In addition, sinusoidal dilatation and Kupffer cell increase were observed. In the cis + gal and cis + sly groups, hepatocytes with pyknotic and karyolytic nuclei were observed in the liver, and hepatocytes with mild pycnotic and karyolytic nuclei were observed in the liver tissue of the cis + gal + sly group.

## 4. Discussion

Cisplatin (cis-diamminedichloroplatinum (II)) is a platinum-based antitumor drug used in the treatment of many types of solid malignancies, including testicular, ovarian, bladder, head and neck, and lung cancer. Although cisplatin fights cancer types, it is life-threatening due to its significant side effects. It shows serious side effects such as nephrotoxicity, hepatotoxicity, and cardiotoxicity. The decrease in GSH level and antioxidant enzymes and increase in MDA due to oxidative stress are seen as the main reason for the toxicity caused by cisplatin [16]. As a result of the reactions of free radicals with guanine bases in DNA, 8OH-dG (8-hydroxy-2′-deoxyguanosine) is formed. Although it is an important factor in the detection of DNA damage, it was observed that the level of 8OH-dG increased as the number of reactive oxygen species formed increased in in vitro and in vivo studies [17]. In an article about cisplatin-induced testicular tissue damage, it was observed that the cisplatin group administered at 5 mg/kg significantly increased the 8OH-dG level on the testicular tissue compared with the control group [18]. In another study, 8OH-dG increased in the urine and kidney tissues of rats receiving cisplatin (15 mg/kg) compared with the control group. In the present study, it was observed that the group receiving cisplatin had a significant increase in the 8OH-dG value in serum, kidney, and liver tissue compared with the control group. It was determined that the groups receiving only cisplatin and gallic acid significantly reduced this increase in liver and kidney tissue caused by the group receiving cisplatin [19]. Malondialdehyde (MDA), the end product of lipid peroxidation, causes cellular damage thanks to the cross-links it forms with the formation of free radicals [20, 21]. In a study on cisplatin, a significant increase was observed in MDA levels in the kidney tissue of rats receiving cisplatin (7 mg/kg) compared with the control group [22]. In a different study, it was observed that rats with cisplatin-induced nephrotoxicity that received cisplatin at 5 mg/kg showed a significant increase in MDA level in kidney tissues compared with the control group. In this study, a significant increase in MDA level was observed in kidney tissues of the group receiving cisplatin compared with the control group. A statistically significant increase was observed in the MDA level of the group receiving cisplatin compared with the control group in the liver tissue. Inadequate antioxidant systems are shown to be the cause of the damage developed by lipid peroxidation [23].

Contributing to antioxidant defense systems in different ways, silymarin inhibits free radicals directly and the enzymes that cause these radicals to form [24]. In a study, when they looked at the therapeutic effects of silymarin and naringin against methotrexate-induced nephrotoxicity, silymarin was administered at 50 mg/kg for seven days. In rats receiving only methotrexate (20 mg/kg), a significant increase in serum urea and creatinine levels was observed, while an increase in MDA was observed in kidney tissues. It was observed that the SOD, CAT, and GPx enzyme activities in the kidney tissues of both silymarin- and naringenin-treated groups increased compared with the methotrexate-treated group. In addition, it was observed that MDA level decreased, and GSH level increased [25]. In a recent study, they examined the protective effect of silymarin against the nephrotoxic and hepatotoxic formation of 5-fluorouracil. 400 mg/kg 5-fluorouracil and 50 mg/kg and 100 mg/kg silymarin were administered. The MDA level in liver tissues was found to be significantly higher in the groups treated with 5-fluorouracil and sly 50 + FU compared with the control group, and it was determined that the GSH level decreased. While the SOD, CAT, and GR activities in the liver were lower in the 5-FU group compared with the control group, it was reported that it increased in a dose-dependent manner of 100 mg/kg in the groups receiving silymarin. In the kidney, MDA level was significantly higher in the 5-FU, control group, while it was lower in the silymarin group, where 100 mg/kg was administered. A decrease was observed in the GSH level compared with the control group. SOD, CAT, and GR enzyme activities were dose-dependently increased in the silymarin-treated groups compared with the 5-fluorouracillin-treated group [26]. In a different study, they investigated the protective effect of silymarin against acute nephrohepatotoxicity caused by mercury. While the MDA and GSH levels measured in the serums caused an increase in MDA in the mercury-treated (5 mg/kg) group, it was reported that they were lower in the silymarin-treated group (200 mg/kg) compared with the mercury-treated group. They stated that GSH levels decreased significantly when mercury was applied compared with the control group, while silymarin increased this decrease. In our current study, the silymarin-treated group significantly decreased the MDA level in the liver tissues compared with the cisplatin-treated group. While there was an increase in CAT activity in the silymarin-treated group compared with the control group, it was not significant. It also showed a significant increase in SOD activity and GSH level [27]. The increase in CAT activity and GSH level was not significant. The increase in SOD activity was found to be significant. This effect of silymarin on antioxidant parameters can be shown as the reason for its free radical scavenging effect thanks to the polyphenolic contents in its structure.

Gallic acid (3,4,5-trihydroxybenzoic acid) is a polyhydroxy phenolic compound. It can be found in natural products such as green tea, grapes, saffron, banana, lemon, pineapple, and strawberry. It is known as a powerful antioxidant, especially due to its free radical scavenging effect [28, 29]. In a study, the protective effect of gallic acid against oxidative stress and liver disorders in rats treated with cyclophosphamide was investigated. Cyclophosphamide was administered as 2 mg/kg, while gallic acid was administered as 20 mg/kg. The creatinine, urea, and bilirubin levels measured on the plasma showed an increase in the cyclophosphamide-treated groups compared with the control group. It was observed that the groups where gallic acid was administered alone or with cyclophosphamide maintained its value compared to those where alone cyclophosphamide was administered. In addition, it was determined that AST and ALT values increased in the cyclophosphamide-treated groups compared with the control group and decreased this increase in the gallic acid-treated groups. It was observed that SOD and CAT activities in liver tissues decreased in the groups treated with cyclophosphamide compared with the control group, and this decrease was improved in the groups where gallic acid was administered together with cyclophosphamide [30]. In a different study, the protective effects of gallic acid on the toxic effects of methotrexate were examined. The MDA level was increased in the groups treated with methotrexate (20 mg/kg) compared with the control group, while the group treated with gallic acid (30 mg/kg) showed no change in the MDA level compared with methotrexate. GSH level decreased in the methotrexate groups, whereas, in the groups treated with gallic acid, GSH increased. While CAT, SOD, and GPx enzyme activities decreased in methotrexate groups compared with the control group, it was observed that gallic acid inhibited this decrease in the groups administered methotrexate and gallic acid [31]. The level was found to be significantly lower. Gallic acid-treated group had a significantly higher CAT activity than the cisplatin-treated group. Gallic acid-treated group had an increase in SOD activity and GSH level in liver tissue compared with cisplatin-treated group, but this increase was not found to be significant. In addition, the level of 8OH-dG in the liver tissue decreased significantly in the gallic acid-treated group compared with the cisplatin-treated group. When we look at the kidney tissues, the MDA level was found to be significantly lower in the gallic acid-treated group compared with the cisplatin-treated group. Compared with the cisplatin-treated group, the gallic acid group significantly increased CAT and SOD activity. Gallic acid increased the GSH level in the kidney tissues compared with the cisplatin-treated group, but this increase was not significant. Gallic acid significantly decreased the 8OH-dG level in kidney tissues compared with the cisplatin-treated group. The polyphenolic content of gallic acid is the most important factor in its antioxidant effect.

The presence of ALT and AST in the blood indicates increased permeability and necrosis of liver cells [32]. Two different studies examined the protective effect of hyperin against cisplatin-induced liver damage. In the study, it was observed that AST and ALT levels in the serum were significantly increased in the cisplatin-treated group compared with the control group [33, 34]. In another study, the protective effect against cisplatin-induced liver and kidney tissue damage was investigated. It was observed that the urea and creatinine levels in the cisplatin-treated group increased significantly in the serum liver tissue compared with the control group in the cisplatin-treated group [35]. Compared with the cisplatin group, the AST level in the liver tissue serum was not significant. Gallic acid-treated group significantly increased ALT level in serum compared with the cisplatin group. Compared with the cisplatin-treated group, gallic acid increased the AST level, and this increase was not significant. The kidney tissues of the cisplatin-treated group showed a decrease in urea level compared with the control group, and this decrease was not significant. The cisplatin-treated group significantly increased the creatinine value compared with the control group. It was observed that the silymarin and gallic acid, which we administered separately, increased the urea level in the serum compared with the cisplatin-treated group, and this increase was not significant. It was stated that creatinine in the serum of the silymarin and gallic acid groups was significantly lower than in the cisplatin group.

Many publications researched the effect of different substances and molecules against the toxic effect of cisplatin in the kidney and liver. In a rat study conducted in this context, undesirable pathological structures were observed in rats where cisplatin was used alone compared with the control group. Again, in the same study, when different molecular structures of gallic acid were applied, it was observed that these structures were normalized [36]. In another study, the effect of pomegranate juice on the liver and kidney tissues of the cisplatin-treated group was investigated. In the study, it was observed that pomegranate juice improved the structural problems caused by cisplatin in the kidney but did not show the same effect in the liver [37]. According to the results obtained in almost all of the studies, the toxic effect of cisplatin in the kidney and liver is clearly seen in the histopathological images [38, 39]. In our current study, parallel results are seen in the performed studies. It has been clearly seen that cisplatin causes undesirable pathological problems in both the liver and kidney. It is clearly seen that silymarin and gallic acid have a curative effect against pathological problems that may occur when used alone or together.

## 5. Conclusion

Cisplatin can cause toxicity by causing physiological changes in many organs. One of the most important pathological effects of cisplatin is that it increases the oxidative stress by increasing reactive oxygen species. Silymarin and gallic acid, together or alone, can protect tissues from toxicity caused by cisplatin by reducing oxidative stress and increasing antioxidant enzymes.

## Figures and Tables

**Table 1:** Comparison of serum GSH, CAT, SOD, and 8OH-dG levels between groups.

Groups	GSH (mmol/L)	CAT (U/L)	SOD (IU/Ml)	8-OHdG (pg/ml)
Control	240.93 ± 27.49^b^	18.39 ± 0.40^a,b^	0.63 ± 0.03^a^	1.24 ± 0.093^c^
Cisplatin	161.48 ± 17.72^c^	15.91 ± 1.43^b^	0.15 ± 0.08^e^	1.86 ± 0.28^a^
Cis + gal	244.59 ± 9.58^b^	17.09 ± 4.68^a,b^	0.30 ± 0.02^c,d,e^	1.31 ± 0.13^b,c^
Cis + sly	214.96 ± 44.56^b^	15.18 ± 1.98^b^	0.28 ± 0.10^d,e^	1.75 ± 0.29^a,b^
Cis + sly + gal	237.21 ± 23.40^b^	22.32 ± 2.56^a^	0.27 ± 0.13^d,e^	1.78 ± 0.31^a,b^
Silymarin	287.32 ± 27.93^a^	20.93 ± 2.92^a,b^	0.58 ± 0.28^a,b^	1.57 ± 0.40^a,b,cb^
Gallic acid	290.21 ± 18.82^a^	17.42 ± 5.25^a,b^	0.52 ± 0.10^a,b,c^	1.43 ± 0.19^a,b,c^
Gal + sly	252.96 ± 31.66^a,b^	22.73 ± 0.83^a^	0.39 ± 0.10^b,c,d^	1.29 ± 0.46^b,c^

a, b, and c: the statistical difference between groups with different letters in the same column is significant (*p* < 0.05). Control: control group; cisplatin: cisplatin group; cis + gal: cisplatin + gallic acid; cis + sly: cisplatin + silymarin; cis + sly + gal: cisplatin + gallic acid + silymarin; silymarin: silymarin group; gallic acid: gallic acid group; gal + sly: gallic acid + silymarin.

**Table 2:** Comparison of GSH, CAT, SOD, MDA, and 8OH-dG levels in liver tissue between groups.

Groups	GSH (mmol/L)	CAT (U/L)	SOD (IU/Ml)	MDA (mmol/L)	8-OHdG (pg/ml)
Control	137.94 ± 21.17^b,c^	27.04 ± 2.64^a^	1.69 ± 0.43^a,b^	0,70 ± 0.22^d^	2.27 ± 0.33^c^
Cisplatin	109.71 ± 10.56^d^	18.83 ± 4.00^b^	1.13 ± 0.37^b^	2.07 ± 0,21^a^	3.91 ± 0.65^a^
Cis + gal	151.22 ± 5.72^a,b^	25.82 ± 1.16^a^	1.90 ± 0.30^a^	1,01 ± 0,13^c^	2.68 ± 0.45^b,c^
Cis + sly	164.59 ± 5.51^a^	24.22 ± 3.09^a,b^	1.90 ± 0.47^a^	1,45 ± 0,41^b^	2.56 ± 0.11^b,c^
Cis + sly + gal	132.41 ± 18.51^b,c,d^	22.38 ± 3.34^a,b^	2.33 ± 0.73^a^	1,12 ± 0,07^c^	2.63 ± 0.72^b,c^
Silymarin	144.76 ± 17.91^a,b,c^	22.43 ± 3.81^a,b^	1.96 ± 0.36^a^	0,99 ± 0,26^c^	2.99 ± 0.22^b,c^
Gallic acid	124.92 ± 16.74^c,d^	26.41 ± 2.46^a^	1.60 ± 0.32^a,b^	1,24 ± 0,09^b,c^	2.73 ± 0.56^b,c^
Gal + sly	136.30 ± 15.06^b,c^	23.53 ± 5.80^a,b^	1.66 ± 0.58^a,b^	1,44 ± 0,19^b^	2.68 ± 0.45^b^

a, b, c, d, and e: the statistical difference between groups with different letters in the same column is significant (*p* < 0.05). Control: control group; cisplatin: cisplatin group; cis + gal: cisplatin + gallic acid; cis + sly: cisplatin + silymarin; cis + sly + gal: cisplatin + gallic acid + silymarin; silymarin: silymarin group; gallic acid: gallic acid group; gal + sly: gallic acid + silymarin.

**Table 3:** Comparison of GSH, CAT, SOD, MDA, and 8OH-dG levels in kidney tissue between groups.

Groups	GSH (mmol/L)	CAT (U/L)	SOD (IU/Ml)	MDA (mmol/L)	8-OHdG (pg/ml)
Control	135.96 ± 30.46^a^	30.60 ± 3.38^a^	2.44 ± 0.27^a,b^	1,36 ± 0,21^b,c,d^	2.19 ± 0.38^e^
Cisplatin	106.10 ± 27.46^a^	20.67 ± 3.45^c^	1.73 ± 0.33^c^	2.30 ± 0,21^a^	4.66 ± 0.47^a^
Cis + gal	167.53 ± 28.96^a^	24.23 ± 2.45^b,c^	2.38 ± 0.38^a,b^	1,13 ± 0,07^d,e^	3.05 ± 0.11^b,c,d^
Cis + sly	162.82 ± 37.01^a^	25.54 ± 5.80^b,c^	2.30 ± 0.47^a,b^	1,57 ± 0,01^b^	3.78 ± 0.46^b^
Cis + sly + gal	128.87 ± 24.80^a^	22.27 ± 1.50^b,c^	2.87 ± 0,41^a^	1,10 ± 0,08^e^	3.30 ± 0.86^b,c,d^
Silymarin	142.57 ± 25.14^a^	23.65 ± 1.26^b,c^	2.40 ± 0.42^a,b^	1,25 ± 0,17^c,d,e^	3.75 ± 0.31^b,c^
Gallic acid	112.04 ± 100.11^a^	26.19 ± 1.76^a,b^	2.43 ± 0.34^a,b^	1,45 ± 0,77^b,c^	2.92 ± 0.25^d,e^
Gal + sly	136.09 ± 13.92^a^	23.16 ± 2.16^b,c^	2.23 ± 0.18^b,c^	1,13 ± 0,27^d,e^	2.95 ± 0.61^c,d,e^

a, b, and c: the statistical difference between groups with different letters in the same column is significant (*p* < 0.05). Control: control group; cisplatin: cisplatin group; cis + gal: cisplatin + gallic acid; cis + sly: cisplatin + silymarin; cis + sly + gal: cisplatin + gallic acid + silymarin; silymarin: silymarin group; gallic acid: gallic acid group; gal + sly: gallic acid + silymarin.

**Table 4:** Intergroup hemogram results.

Groups	WBC	RBC	HGB	HCT	PLT
Control	4.13 ± 1.33^c^	6.89 ± 0.49^d^	13.72 ± 1.13^c^	48.40 ± 4.40^a^	1062.25 ± 133.^a^
Cisplatin	7.56 ± 3.70^b,c^	8.26 ± 0.67^a,b,c^	16.56 ± 1.06^a^	50.46 ± 2.37^a^	686.33 ± 235.92^b^
Cis + gal	6.58 ± 2.15^b,c^	8.44 ± 0.85^a,b^	16.80 ± 1.41^a^	53.33 ± 3.26^a^	949.33 ± 134.79^a,b^
Cis + sly	10.65 ± 1.18^a,b^	8.42 ± 0.74^a,b^	16.35 ± 1.48^a,b^	53.90 ± 6.08^a^	1030.50 ± 219.91^ab^
Cis + sly + gal	14.36 ± 3.55^a^	8.71 ± 0.75^a^	16.60 ± 1.44^a^	55.26 ± 3.70^a^	806.00 ± 271.06^a,b^
Silymarin	8.11^b,c^	7.29^c,d^	14.40^b,c^	50.50^a^	893.00^a,b^
Gallic acid	7.68 ± 1.46^b,c^	7.52 ± 0.21^b,c,d^	14.93 ± 0.97^a,b,c^	52.46 ± 3.91^a^	816.00 ± 176.90^a,b^
Gal + sly	8.02 ± 1.58^b,c^	7.39 ± 0.23^b,c,d^	15.63 ± 0.32^a,b,c^	52.93 ± 1.76^a^	789.33 ± 89.114^a,b^

a, b, and c: the statistical difference between groups with different letters in the same column is significant (*p* < 0.05). Control: control group; cisplatin: cisplatin group; cis + gal: cisplatin + gallic acid; cis + sly: cisplatin + silymarin; cis + sly + gal: cisplatin + gallic acid + silymarin; silymarin: silymarin group; gallic acid: gallic acid group; gal + sly: gallic acid + silymarin.

**Table 5:** Intergroup biochemistry results.

Groups	AST (IU/L)	ALT (IU/L)	Urea (mg/dl)	Creati̇ni̇ne (mg/dl)	Albumin (g/dl)	Globulin (g/dl)	Total protein (g/dl)
Control	108.20 ± 12.47^a,b^	32.00 ± 3.05^a^	53.80 ± 6.97^c,d^	0.48 ± 0.04^b^	28.40 ± 2.7^a,b^	28.60 ± 3.05^b^	57.00 ± 5.29^a^
Cisplatin	124.67 ± 6.65^a^	35.67 ± 3.78^a^	74.67 ± 6.65^a^	2.41 ± 0.37^a^	26.33 ± 1.15^a,b^	29.00 ± 3.60^b^	55.33 ± 4.50^a^
Cis + gal	94.00 ± 24.52^b^	23.60 ± 7.60^a^	57.50 ± 3.69^c,d^	0.59 ± 0.08^b^	25.00 ± 1.58^b^	31.20 ± 6.83^a,b^	56.20 ± 6.09^a^
Cis + sly	96.40 ± 10.47^a,b^	30.60 ± 8.38^a^	62.20 ± 9.52^b,c^	0.62 ± 0.22^b^	26.80 ± 1.78^a,b^	29.60 ± 1.34^a,b^	56.40 ± 3.05^a^
Cis + sly + gal	110.80 ± 38.88^a,b^	35.80 ± 12.91^a^	70.80 ± 13.44^a,b^	0.72 ± 0.36^b^	26.00 ± 3.60^a,b^	34.60 ± 4.03^a^	60.60 ± 4.98^a^
Silymarin	89.83 ± 10.96^b^	33.17 ± 6.96^a^	40.83 ± 5.63^e^	0.44 ± 0.02^b^	29.17 ± 0.75^a^	29.67 ± 1.03^a,b^	58.83 ± 0.98^a^
Gallic acid	95.00 ± 9.21^b^	32.00 ± 5.29^a^	48.50 ± 8.42^d,e^	0.45 ± 0.04^b^	28.50 ± 0.57^a,b^	29.75 ± 0.50^a,b^	58.25 ± 0.95^a^
Gal + sly	100.50 ± 5.91^a,b^	29.75 ± 9.77^a^	56.67 ± 2.51^c,d^	0.49 ± 0.05^b^	29.25 ± 3.59^a^	32.00 ± 1.15^a,b^	61.25 ± 4.34^a^

a, b, and c: the statistical difference between groups with different letters in the same column is significant (*p* < 0.05). Control: control group; cisplatin: cisplatin group; cis + gal: cisplatin + gallic acid; cis + sly: cisplatin + silymarin; cis + sly + gal: cisplatin + gallic acid + silymarin; silymarin: silymarin group; gallic acid: gallic acid group; gal + sly: gallic acid + silymarin.

## Data Availability

The data used to support the findings of this study are included within the article.
